# Bias in algorithms of AI systems developed for COVID-19: A scoping review

**DOI:** 10.1007/s11673-022-10200-z

**Published:** 2022-07-20

**Authors:** Janet Delgado, Alicia de Manuel, Iris Parra, Cristian Moyano, Jon Rueda, Ariel Guersenzvaig, Txetxu Ausin, Maite Cruz, David Casacuberta, Angel Puyol

**Affiliations:** 1 Department of Philosophy 1, Faculty of Philosophy, University of Granada, Granada, Spain; 2 Department of Philosophy, Universitat Autònoma de Barcelona, Barcelona, Spain; 3 FiloLab Scientific Unit of Excellence of the University of Granada, Granada, Spain; 4 Elisava School of Design and Engineering, UVIC-UCC, Barcelona, Spain; 5 Institute for Philosophy of the Spanish National Research Council (CSIC), Madrid, Spain; 6 Andalusian School of Public Health (EASP), Granada, Spain

**Keywords:** artificial intelligence, bias, digital contact tracing, COVID-19, patient risk prediction

## Abstract

To analyze which ethically relevant biases have been identified by academic literature in artificial intelligence (AI) algorithms developed either for patient risk prediction and triage, or for contact tracing to deal with the COVID-19 pandemic. Additionally, to specifically investigate whether the role of social determinants of health (SDOH) have been considered in these AI developments or not. We conducted a scoping review of the literature, which covered publications from March 2020 to April 2021. ​Studies mentioning biases on AI algorithms developed for contact tracing and medical triage or risk prediction regarding COVID-19 were included. From 1054 identified articles, 20 studies were finally included. We propose a typology of biases identified in the literature based on bias, limitations and other ethical issues in both areas of analysis. Results on health disparities and SDOH were classified into five categories: racial disparities, biased data, socio-economic disparities, unequal accessibility and workforce, and information communication. SDOH needs to be considered in the clinical context, where they still seem underestimated. Epidemiological conditions depend on geographic location, so the use of local data in studies to develop international solutions may increase some biases. Gender bias was not specifically addressed in the articles included. The main biases are related to data collection and management. Ethical problems related to privacy, consent, and lack of regulation have been identified in contact tracing while some bias-related health inequalities have been highlighted. There is a need for further research focusing on SDOH and these specific AI apps.

## Background

During the COVID-19 pandemic, one of the most widespread measures adopted to control, minimize, and mitigate the impact of COVID-19 was the development of mobile apps that use a variety of technologies to log information used to identify the spread of the disease, physical symptoms of individuals, and possible close contacts. Digital contact tracing (DCT) via smartphone apps was established as a new public-health intervention in many countries in 2020 to reduce the levels of COVID-19 transmission (Colizza et al. 2021). Nevertheless, DCT systems may perpetuate some biases that influence the results obtained, while raising security and privacy concerns (Bengio et al. 2020; Sun et al. 2021).

Another big effort has been put into developing decision-making support devices to help clinicians at the bedside. There is an increasing multiplicity of artificial intelligence (AI) systems and algorithms focused on COVID-19 early detection in risk patients and their prognosis (Jamshidi et al. 2020). Studies prove that these novel technologies support medical triage in those circumstances where healthcare resources are scarce. Still, their results show some limitations due to technical issues or regarding social, cultural, and economical aspects that have been overlooked.

A general definition of bias would be a “strong inclination either in favor or against something” (Moseley 2021). By algorithmic biases in AI, we refer to systematic errors in a computer system, with a consequent deviation from the expected prediction behavior of an AI tool (Amann et al. 2020). These deviations can come either from the design of the algorithm or from previous data collection, coding, and selection. Both options have to be taken into account while analysing possible limitations and poor performances of AI outcomes in the areas stated above. Regarding the data, biases emerge mainly from the data used to train the algorithm —through sampled data or data in which societal biases already existed (Tsamados et al. 2022). Previous databases can generate unfair results if there is no representativity of the population diversity or if some segment is over-represented while others are under-represented (Tsamados et al. 2022). Design biases are those associated with previous conceptual decisions made by the providers to create the machine learning (ML) system which may generate results that systemically affect a segment of the population.

While other reviews analyse the application of AI designed for COVID-19 (Guo et al. 2021), our intention is to focus this one in two specific topics: biases exclusively in AI systems developed for 1) DCT and 2) medical triage regarding COVID-19, as they have been two of the most widely developed automatized systems during the early phases of the pandemic and they need to be evaluated. In addition, although certain social health conditions have been discussed in general terms, social determinants of health (SDOH) are explicitly addressed in neither clinical research nor technical development of apps. Consequently, we hypothesize that there may be a lack of qualitative data analysis in their application, which causes an overlook of health disparities and SDOH and that have effects on people’s health. Clinical research and app development research are mostly focused on biological-only data, which not only may retain biases and exacerbate health inequalities (Röösli, Rice, and Hernandez-Boussard 2021) but also underestimate social-related biases in their analysis.

Thus, in this scoping review we aim to summarize some of the ethically relevant types of bias that have already been identified in literature in AI systems developed for DCT, and for patient risk prediction (PRP) or medical triage to deal with COVID-19 pandemic. In addition, a secondary goal is to analyse if there is any relationship pointed out by previous literature between the biases and social determinants of health in AI systems and algorithms developed for COVID-19.

## Materials and Methods

A scoping review can be a useful approach when the information on a topic has not been comprehensively reviewed, which is the case of research themes. This scoping review followed the recommended five-step framework for scoping reviews (Arksey and O’Malley 2005; Munn et al. 2018; Pham et al. 2014; Tricco et al. 2016). The research questions were: What are the biases identified in the literature in AI systems or algorithms developed for COVID-19 triage or PRP and DCT? Do researchers and engineers consider the SDOH? Do they relate these biases to possible health disparities?

### Search Strategy and Selection Process

We carried out the search strategy in Pubmed, Medline, CINAHL, Scopus, Wiley Online Library, WOS, and Arxiv.org, between the March 1 and the April 7, 2021. The search strategy was initially developed in Pubmed and then adapted to the other databases (Table 1).

**Table 1:** Search strategy

#1 Bias	
#2 Bias OR Social determinants of health	
#3 Algorithmic injustice OR prejudice OR discrimination	
#4 Artificial Intelligence OR Machine Learning OR AI OR Algorithms	
#5 Artificial Intelligence OR Machine Learning OR AI OR Algorithms OR Deep Learning	
#6 Smartphone OR Geolocation OR App OR Mobile Phone	
#7 Contact tracing app	
#8 Social determinants of health OR Income OR social protection OR Education OR Unemployment OR job insecurity OR Working life conditions OR Food insecurity OR Housing OR basic amenities OR environment OR Early childhood development OR Social inclusion OR non-discrimination OR Structural conflict OR health services	
#9 Covid-19 OR Coronavirus OR SARS-CoV-2	
#10. #2 AND #4 AND #9	
#11. #1 AND #4 AND # 8 AND #9	
#12. #3 AND #5 AND #9	
#13. #1 AND #6 AND #9	
#14. #1 AND #7 AND #9	

A manual search was performed to retrieve additional relevant documents not located by the electronic search. Studies fulfilling the criteria shown in Table 2 were eligible. Following the removal of duplicates, two reviewers screened all studies by title and abstract. Full-text articles were obtained for full-text screen, and each one was read by two reviewers, based on the inclusion and exclusion criteria. Discrepancies were discussed and a consensus was reached. When consensus was not possible, all the four reviewers made a decision together.

**Table 2:** Inclusion and exclusion criteria

Inclusion criteria	
Type of publication	Studies with quantitative methodology, mixed methodology, qualitative, interventions, narrative reviews, scoping reviews, systematic reviews/meta-analysis, randomized clinical trials, editorials, and letters to editor.
Subject or domain being studied	Articles focused on or with mention of biases, algorithms, or AI systems developed for COVID-19, used in triage, early detection of risk patients, and contact tracing.
Language	English or Spanish.
Participant/population	AI systems or algorithms developed for any population group.
Intervention	Any intervention related to our condition.
Date	Articles published or accepted between March 2020 and April 2021.
Exclusion criteria	
Type of publication	Books and theses, not accepted for publication preprints, conferences, and abstracts.
Subject or domain being studied	Articles about COVID-19 which do not include any algorithm or AI system or which do not mention biases. Articles focused on bias, but not on AI systems for COVID-19. Articles about other topics than triage, risk prediction, or contact tracing (for instance, vaccines, clinical trials).
Language	Different from English or Spanish.
Participants/population	No exclusion criteria.
Intervention	No exclusion criteria.
Date	Articles prior to 2020.

### Data Extraction and Data Synthesis

Data extraction was undertaken by the four reviewers using an agreed template designed ad hoc. We created a table gathering the main characteristics and results of the studies to collect information from the data extraction. Finally, we developed a narrative synthesis of the main findings.

## Results

**Fig. 1:**
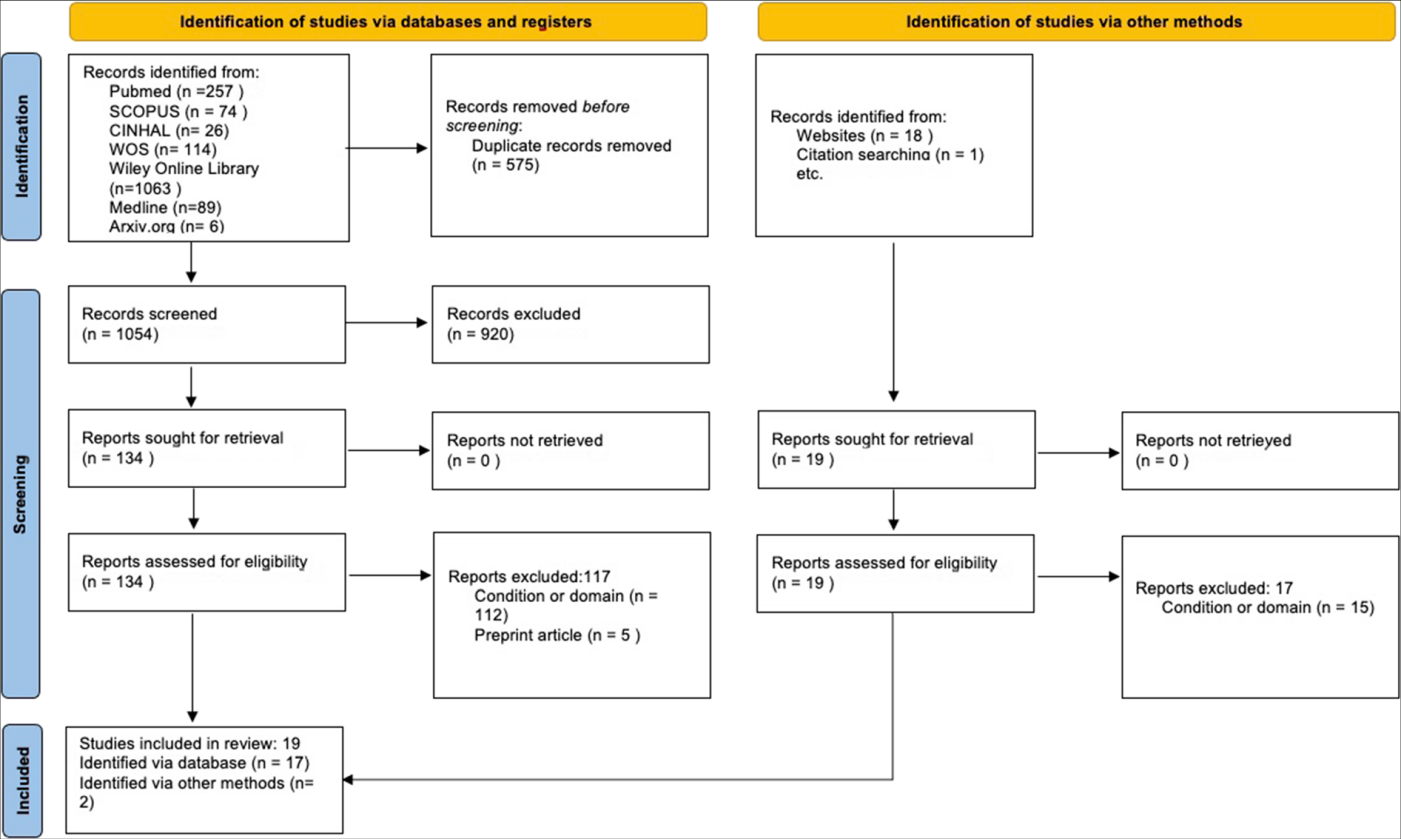
Scoping Review Flow Diagram following the PRISMA 2020 statement proposed by Page et al. (2021)

After screening by title and abstract, out of the 1054 identified articles after eliminating duplicates, 134 were included for full-text reading, and sixteen met the criteria for the extraction of relevant information. A manual search provided four additional studies; thus, twenty were finally eligible for inclusion (Fig. 1).

Of those selected, we found ten narrative reviews (Grantz et al. 2020; Klingwort and Schnell 2020; Mali and Pratap 2020; Malik et al. 2021; Marabelli, Vaast, and Li 2021; Mbunge 2020; Park, Choi, and Ko 2020; Roche 2020; Röösli, Rice, and Hernandez-Boussard 2021; Scott and Coiera 2020; Shachar, Gerke, and Adashi 2020), five original articles (Casiraghi et al. 2020; Hisada et al. 2020; Marabelli, Vaast, and Li 2021; Moss and Metcalf 2020 ; Ravizza et al. 2021), two case studies (Gulliver, Fahmi, and Abramson 2020; Sáez et al. 2021), two systematic reviews (Mbunge et al. 2020; Wynants et al. 2020), and one rapid review (Anglemyer et al. 2020). For countries, we found five articles from the United States (Grantz et al. 2020; Marabelli, Vaast, and Li 2021; Moss and Metcalf 2020; Röösli, Rice, and Hernandez-Boussard 2021; Shachar, Gerke, and Adashi 2020), one from the United States and South-Korea (Park, Choi, and Ko 2020), two from Australia (Scott and Coiera 2020; Gulliver, Fahmi, and Abramson 2020), Italy (Casiraghi et al. 2020; Ravizza et al. 2021), India (Malik et al. 2021; Mali and Pratap 2020) and Swaziland (Mbunge 2020; Mbunge et al. 2020), and one from The Netherlands (Wynants et al. 2020), Spain (Sáez et al. 2021), Germany (Klingwort and Schnell 2020), Japan (Hisada et al. 2020), New Zealand (Anglemyer et al. 2020), and Canada (Roche 2020). While four studies addressed both triage or PRP and DCT, three studies addressed only triage or PRP, and thirteen only DCT.

Table 3 summarizes the different apps identified in this study. We use the term “bias” to refer to the systematic errors in a computer system causing an inclination or prejudice for or against a person or a group of people that can be considered to be unfair and cause a deviation from the expected prediction behavior of an AI tool, and “limitations” to refer to facts or situations that allow only some actions and make others impossible.

**Table 3:** DCTApps identified in the scoping review

App	Type	Technology	Reference
TraceTogether (Singapour)	Contact Tracing	Bluetooth (BlueTrace protocol)	(Roche 2020)
The-Corona-Warn-App (Germany)	Contact Tracing	Control smartphone	(Mbunge 2020)
COVIDSafe (Australia)	Outbreak Response	Bluetooth (BlueTrace protocol)	(Gulliver, Fahmi, and Abramson 2020)
Stopp Corona (Austria)	Contact Tracing	Bluetooth	(Mbunge 2020)
BeAware App (Bahrain)	Contact Tracing	Bluetooth and Global System for Mobile Communications technology	(Mbunge et al. 2020)
HaMagen (Israel)	Contact Tracing	Bluetooth and GPS	(Mbunge et al. 2020)
bStayHomeSafe (China)	Contact Tracing	Bluetooth, GPS, and WiFi	(Mbunge 2020)
CoronaApp (Colombia)	Contact Tracing	Global Positioning System	(Mbunge et al. 2020)
Aarogya Setu (India)	Contact Tracing	Global Positioning System	(Mbunge 2020)
GH COVID-19 (Ghana)	Outbreak Response	Global Positioning System and GIS	(Mbunge et al. 2020)
CoronaMadrid (Spain)	Symptom tracking	Global Positioning System	(Mbunge 2020)
Social Monitoring (Russia)	Quarantine compliance	Global Positioning System	(Mbunge et al. 2020)
Yahoo! JAPAN App (Japan)	Contact Tracing	WSSCI	(Hisada et al. 2020)
StopCovid (France)	Contact Tracing	Bluetooth	(Roche 2020)
STOPV (France)	Contact Tracing	Global Positioning System, Semantic Data, Epidemiological Data and Test Results	(Roche 2020)
Private Kit: Safe Paths (USA)	Contact Tracing	Global Positioning System	(Roche 2020)
Covid Alert (Canada)	Contact Tracing	Bluetooth	(Roche 2020)

### AI Systems Developed for Triage and PRP

#### Bias

One of the most relevant aspects addressed in the literature is data-related biases. According to a systematic review (SR) of COVID-19 prognostic and risk prediction methods (Wynants et al. 2020), there is a high risk of bias in the studies included due to a poor description of the population, which raises concerns about the reliability of their predictions when applied to clinical practice. An immediate exchange of well-documented individual participant data from COVID-19 studies is needed to develop more rigorous prediction models and validate the existing ones through collaborative efforts.

Our results identified different types of data-related bias:

1. Data source variability contributes to bias in distributed research networks of COVID-19 data sharing (Sáez et al. 2021), and they play an important role in data quality. The case study reported by Sáez et al. (2021) shows the limitations that multisource variability may have for COVID-19 machine learning (ML) research on international distributed research networks. They used the nCov2019 dataset, including patient-level data from several countries, to discover and classify severity subgroups, dividing them into six types: 1) mild disease with no comorbidity, 2) elderly + severe pulmonary disease + comorbidity, 3) middle-aged + severe pulmonary disease + no comorbidity, 4) elderly + mild disease + no comorbidity, 5) elderly + severe systemic disease + comorbidity, 6) elderly + severe pulmonary disease + heart failure. The problem appears when this division is conditioned by the data’s country of origin. Groups 1 and 4 data were collected in China and groups 2, 3, 5, and 6 in the Philippines. In the last case, data came from the COVID-19 tracker, owned by the Department of Health of the Republic of Philippines; in the case of China, data came mostly from patient reports. Due to these variations in the sources, results show some inconsistencies, limiting the model. Potential biases of multisource variability for ML can be generalized in large cross-border distributed research networks. How can we prevent such biases? Sáez et al. (2021) propose 1) a routine assessment of the variability among data sources in ML and statistical methodologies to potentially reduce biases or extra costs, 2) a complete data quality assessment, incorporating source and temporal variability, 3) reporting data quality and its impacts as a routine practice in publications, and 4) building consciousness about data quality and variability.
2. Casiraghi et al. (2020) developed an explainable PRP model for COVID-19 risk assessment aimed to avoid data bias. Their model was designed to be used in emergency departments for an early assessment of PRP in COVID-19 patients, integrating clinical, laboratory, and radiological data. The study carried out a comparative evaluation of different imputation techniques to manage the problem of missing data in the prediction for COVID-19 patients. However, the lack of a shared dataset hindered an objective comparative evaluation with the best models (Casiraghi et al. 2020).
3. There are biases in COVID-19 prediction models due to unrepresentative data samples, high probability of model overfitting, imprecise information on the study populations, and the use of a model unsuited to the task (Röösli, Rice, and Hernandez-Boussard 2021). Models developed in elite and affluent academic health systems that are not representative of the general population lack external validity (Röösli, Rice, and Hernandez-Boussard 2021).
4. The quick development of AI systems carries great risk due to skewed training data, lack of reproducibility, and lack of a regulated COVID-19 data resource (Röösli, Rice, and Hernandez-Boussard 2021). Without comprehensive bias mitigation strategies, this can exacerbate existing health disparities. “The source code of any AI model should be shared publicly to ensure that the models can be widely applied, generalized, and transparently compared” (Röösli, Rice, and Hernandez-Boussard 2021, 191).

#### Limitations

A sufficient amount of high-quality data is crucial for the successful implementation of AI in COVID-19 management. Designing practical AI-based algorithms is challenging because of the huge and complex data that emerge as a consequence of the varied manifestations of the COVID-19 infection, ranging from asymptomatic to severe clinical disease (Malik et al. 2021). Moreover, the principal obstacle to implement these systems in the clinical context is the regulation of the data exchange obtained by the AI application. Additionally, AI-based algorithms can offer a binary answer to a specific question about the disease in context but cannot offer alternative predictions (Malik et al. 2021). Finally, it is necessary to consider how meaningful and in-depth data can be generated at every point of healthcare activity (Malik et al. 2021).

#### Other ethical issues

Transparency in AI algorithms is essential to understand predictions and target populations, unrecognized biases, class imbalance problems, and their capacity to generalize emerging technologies across hospital settings and populations (Röösli, Rice, and Hernandez-Boussard 2021). To ensure that models can be broadly applied, generalized, and compared, the source code of an AI system should be shared publicly, and regulatory frameworks should be created to facilitate data sharing (Röösli, Rice, and Hernandez-Boussard 2021).

### AI Systems Developed for DCT

#### Bias

1. Uncontrolled application development could generate inadequate data collection and biases due to the loss of some data or an insufficient frequency of monitoring, which can lead to an inability to compare data collected from different regions (Ravizza et al. 2021). Although it does not affect the core functionality of the app, it can influence further use of the collected data: most ML models have relied on Chinese data, which can limit scalability to other populations (Scott and Coiera 2020).
2. The media alter the nature of searches, producing biases in areas of potential clusters. Whenever the media report a location of a positive COVID-19 patient, many people who are close to the informed location ask for additional information related to COVID-19 (Hisada et al. 2020).
3. DCT Applications (DCTApps) pose a high risk of discrimination, especially to affected people (Mbunge 2020). Specifically, internet-of-things (IoT) based DCTApps collect data from the entire population in real time, which is later analysed to map COVID-19 hotspots. Such data include ethnic information, demographic details, and socioeconomic status, which can influence the allocation and distribution of COVID-19 resources, potentially leading to discrimination.
4. False negatives are an obstacle and may be deliberately generated because infected people do not want to reveal their true status (Klingwort and Schnell 2020). To overcome this problem, the detection of relevant contacts should be refined as the issue is fundamentally a problem of microscale spatial analysis. Applications must develop the microgeographic analytical capability to specify what kind of proximity constitutes a sufficient contagion risk to trigger a notification (Roche 2020).

#### Limitations and Technical Problems

1. Accuracy: The most widely proposed type of COVID-19 application uses Bluetooth signals to track encounters with people diagnosed as infected after the encounter; the accuracy of automatic DCTApps suffers from Bluetooth-based measurement errors (Klingwort and Schnell 2020). These errors are due to the devices’ different signal strengths and the fact that signal is not transmitted in all directions. Characteristics of the physical environment (windows, walls, or doors) can affect the range of discoverable devices. In addition to the four efficiency conditions (mass adoption, well-equipped population, numerous diagnostic tests, and fair and transparent uses), these monitoring applications have many reliability limitations, especially in the Bluetooth reading forecast and the calibration (Roche 2020). This can add noise and produce many false positives.
2. Data-related problems: Most applications have not reached operational maturity (Scott and Coiera 2020), and their effectiveness has not been proved (Anglemyer et al. 2020). Even modelling studies provide low-certainty evidence of a reduction in secondary cases if CT is used together with other public health measures such as self-isolation. Cohort studies provide very low-certainty evidence that digital DCT may produce more reliable counts of contacts and reduce time to complete DCT (Anglemyer et al. 2020). The performance of emerging technologies is not yet stable in account of the lack of availability of a sufficient COVID-19 dataset, the inconsistency of some of the available datasets, the non-aggregation of the dataset, and missing data and noise (Mbunge et al. 2020).

DCTApps may use the IoT to transfer data to national health systems. However, they are not globally standardized, and they face a lot of problems based on interoperability (heterogeneity of connection standards and communication protocols, data semantics, formats, different operating systems, and programming languages). Consequently, each country has developed its own app. Data formats and structures should be standardized to avoid noise, prevent incomplete data, and improve quality (Mbunge 2020; Mbunge et al. 2020). Determining a standardized list of data, symptoms, clinical signs, risk factors, and comorbidities associated with coronavirus can contribute to ensuring compatibility of databases between regions and countries and to improving interoperability (Ravizza et al., 2021).

DCT becomes less effective when dealing with asymptomatic individuals, since symptom checkers and apps rely on pulse, temperature, and sleeping patterns (Hellewell et al. 2020, cited in Mbunge 2020). Due to built-in privacy mechanisms, the resulting data for scientific research based on these applications are limited to counts of positive or negative encounters from selective populations, where the odds of encounters cannot be calculated (Klingwort and Schnell 2020).

#### Other Ethical Issues

1. Privacy concerns: The use of DCTApps raises ethical, legal, security, and privacy concerns (Roche 2020). To be acceptable, this interference with fundamental rights must be justified, reasonable, proportionate, and politically consensual. DCTApps provide little or no privacy to infected people and require them to disclose their data, raising difficult issues of consent, privacy, ethics, and trade-offs between public and private goods (Scott and Coiera 2020).

DCTApps violate the security, confidentiality, integrity, and data availability of COVID-19 patients and contact persons, which can sometimes cause mental health issues like stress, anxiety, or depression (Mbunge, 2020). Apps like TraceTogether, COVIDSafe, or BeAware support access to multiple data access points and the monitoring and surveillance of infected or isolated people, which threatens the security of public health data, and may be a violation of privacy (Mbunge 2020).

The study of Park, Choi, and Ko (2020) in South Korea recreates privacy-related problems. In the face of the outbreak of COVID-19, the South Korean Ministry of Health and Welfare (MOHW) made the following information available to the public on the Internet or through a press release: 1) the route and means of transportation of infected people, 2) the medical institutions that treated the infected people, 3) and the health status of those in contact with an infected person. In addition to these items, sex, nationality, and age of those infected were available, although their names were not disclosed. Some municipal and local governments went further and provided very detailed routes and the names of restaurants, shops, and other commercial premises that infected people visited. The locations of the infected people attracted extensive news coverage. Some of these people were affected by an unwanted invasion of privacy and were even the subject of public disdain. Restaurants, shops, and other commercial venues that infected people visited experienced dramatic economic losses. Concerns were raised regarding the uneven scope and granularity of disclosures by municipal and local governments. Korea’s National Human Rights Commission issued a recommendation to ameliorate privacy concerns, suggesting that the revelation of exceedingly detailed information was unwarranted. Instead of disclosing data to the public, information could be used to sanitize establishments, potentially avoiding stigma and business decline. That is, instead of publicly revealing the precise locations of an infected individual, less granular data could be revealed, with the same effect on tracking and quarantine (Park, Choi, and Ko 2020).

The correlation of data, the exchange of information, and the ability to extract information from different entry points contribute to the increasing fragility of the anonymization of data. This anonymization is even more fragile when information is collected over time and through data cross-referencing (Roche 2020). The deactivation of DCTApps must be programmed so that monitoring does not continue beyond the health emergency and is not tacitly established as standard practice. Otherwise, risks of mass surveillance could arise.

2. Lack of regulation: There are no specific regulations for DCTApps. However, their use of data, access, or privacy has been adapted to international, national, and state laws such as the European Union’s General Data Protection Regulation (GDPR) and the California Consumer Privacy Act (CCPA). These legal frameworks can be adapted to help address concerns about privacy, human rights, due process, and equality (Shachar, Gerke, and Adashi 2020). In certain countries, like the United States, the lack of state regulation makes it more difficult to guarantee that these applications follow ethical standards (Shachar, Gerke, and Adashi 2020), and there are no global WHO guidelines on health data shared and transmitted via 5G technology (Mbunge et al. 2020). Though some countries’ regulations protect citizens better, potential “digital scars” are left in society as long as the governments and private institutions continue having long-term and unlimited access to citizens’ data for surveillance purposes.

3. Consent: The efficacy of DCTApp depends on the level of population uptake, its ability to accurately detect infectious contacts, and the extent of adherence to self-isolation by notified contacts (Scott and Coiera 2020). DCT must be handled with care: these technological solutions are proposed as the only tool available to ensure a process of deconfinement, a requirement that would make it a sine qua non condition accessible to police control. The risks of seeing such an established form of socio-spatial “triage” and patients and certain categories of the ostracized population are huge (Roche 2020).

Although DCTApps use WiFi, GPS, or Bluetooth protocols to monitor people’s movement, users have the right to opt-out and configure their devices, jeopardizing the monitorization of positive cases (Mbunge 2020). DCTApp should allow people to practice withdrawal of consent (Mbunge 2020), as problematic uses of technologies may well remain once the pandemic is over. This can potentially advantage powerful groups that can obtain financial and political benefits from perpetuating the use of IT surveillance while having questionable effects on society (Marabelli, Vaast, and Li 2021).

Privacy issues related to forcing a population to use an app can lead to much lower coverage rates (Klingwort and Schnell 2020). However, we find opposite scenarios in countries that have not developed any specific app. Brazil, for example, has increased its technological surveillance in order to minimize the COVID-19 transmission chain (Mbunge et al. 2020). This enforcement of massive surveillance can raise issues about power, abuse, and data exploitation.

## Health Disparities and Social Determinants of Health in AI Systems Developed for Triage and DCT for COVID-19

### Racial Disparities

Health disparities are related to the emergence of biases in ML systems in the U.S. context where Black and Latinx communities have been the most severely affected by COVID-19 (Moss and Metcalf 2020). This is due to long-standing disparities in health outcomes for these communities, the impact of environmental determinants of health, and the disproportionate number of workers whose jobs do not allow them to stay at home (Moss and Metcalf 2020).

### Biased Data

The reliance on AI may create a false sense of objectivity and fairness (Röösli, Rice, and Hernandez-Boussard 2021). The pervasiveness of biases is a failure to develop mitigation strategies and has exacerbated the risk of existing health disparities, hindering the adoption of other tools that could actually improve patient outcomes. As an example, the Medical Information Mart for Intensive Care (MIMIC) is a publicly available, de-identified, and broadly studied dataset for critical-care patients. A MIMIC-equivalent for COVID-19 from diverse data sources could incentivize urgently needed data sharing and interoperability to enable diverse, population-based tailored therapy—a step that could decisively reduce biases and disparities in healthcare while bolstering clinical judgement and decision-making. One of the main methodological problems is the selection process (Klingwort and Schnell 2020). The sample of the population using the application will not be random, and subpopulations with a higher prevalence of undetected infections will likely have lower coverage. In addition, models that include comorbidities associated with worse outcomes in COVID-19 may perpetuate structural biases that have led to historically disadvantaged groups disproportionately suffering those comorbidities. To avoid further harm to minority groups already most affected by COVID-19, resource allocation models must go beyond a utilitarian foundation and must be able to identify needs amongst these patients (Moss and Metcalf 2020).

### Socio-Economic Disparities

In DCT, the ability to make use of notifications to minimize one’s own risk by self-quarantining is far too dependent on one’s personal wealth and capacity to afford to stay at home (Moss and Metcalf 2020). DCTApps’ designers must be attuned to the context of social life in which such systems can produce harmful, difficult-to-foresee effects that replicate or amplify pre-existing inequalities. Attending the contextual use of such a system could collectivize risk by identifying and emphasizing the necessary forms of social support for self-quarantine and medical care: adequate sick leave and quarantine leave policies, robust testing, and the economic relief that targets individual workers over large companies.

During the pandemic, ML has been involved in the production and distribution of risk through society (Moss and Metcalf 2020), generating risks and its uneven distribution in society. Many of the predictive surveillance algorithms used in DCT control focus attention on populations where bias is very present, especially in highly racialized or lower-income populations (Moss and Metcalf 2020). In this sense, ML can be epidemiologically effective, while unethical.

### Unequal Accessibility

AI-based global health initiative is recommended, since AI-based approaches may not be accessible in countries with limited resources (Malik et al. 2021). Regarding socio-economic disparities and the digital gap, the lack of population coverage can leave certain populations at risk (Ravizza et al. 2021). Digital solutions can exacerbate existing disparities between those who do not have access to smartphones or who live in areas without connectivity, because of ethnicity, socio-economic status, or age (Anglemyer et al. 2020), with equity implications for at-risk populations with poor access to the Internet and digital technology. Digital deserts or data poverty in certain geographical areas are concerning, especially because the effectiveness of DCTApps depends on their massive voluntary adoption and a systematic screening (Roche 2020). Across country borders, the health gap and inequalities in healthcare pose a problem for the integration of emerging technologies. Even in developed countries, risk groups may not have access to broadband, smartphones, or wearable technology. For a community to benefit from this technology, most people need to be equipped with mobile devices. This applies to only eighty per cent of the US population, sixty-five per cent of Russians, and forty-five per cent of Brazilians (Marabelli, Vaast, and Li 2021). Children, elderly, or individuals with fewer resources are excluded from the stored information (Grantz et al. 2020).

### Workforce and Information and Communication Technologies Infrastructure

Developing an app and maintaining the system requires a specific workforce and a consistent information and communication technologies (ICT) infrastructure that may be lacking. Some countries may struggle with the technological infrastructure, especially in countries with a high incidence (e.g., Chad or the Central African Republic) where ICT infrastructures are very poor. These factors can hinder the development of technological innovation policies as part of their response to COVID-19 (Mbunge 2020).

## Discussion

This is the first study to offer a categorization by typology of the main biases, limitations, and related ethical issues identified in current scholarship on AI systems developed for triage or PRP and DCTApps during COVID-19. Many papers have already stressed the matter of bias in artificial intelligence in different areas, such as the content and use of websites (Baeza-Yates, 2018), the process of recruitment (Dastin 2018), the racial bias in assessment in criminal sentences (Angwin et al. 2016), or the racial bias in facial recognition (Buolamwini and Gebru 2018), among others. However, the specific focus on the role of SDOH in the emergence of bias in these systems and its analysis is a novel contribution that can enrich the perspective of technological development for future crises.

One of the main findings from this review is that while references to “health disparities” are relatively more frequent in the study of AI systems, references to “SDOH” are rather uncommon. We may also point out that definitions and terminologies vary from one author to another, which has made it more difficult to identify and systematize them. Based on the results, we argue that SDOH and health disparities are rarely taken into account in triage and PRP studies and are mostly related to DCTApps. These findings suggest that SDOH are undervalued in a clinical context and need to be given more consideration.

Our review shows that data are geographically dependent and that their use for training across regions and countries results in biases in the outcomes. The use of local data to develop international solutions can increase biases in other local populations due to epidemiological differences. Previous studies have suggested that increasing the diversity of data sets from different populations and geographical regions and demonstrating the reproducibility of AI-based algorithms in different settings would be required if the usage of AI tools is going to be generalized (Chen and See 2020; Nagendran et al. 2020; Zou and Schiebinger 2021). As some authors have pointed out, the shared use of public databases might facilitate future international analysis aimed at noticing the interspatial diversity and multiple SDOH factors affecting under-represented groups (Hendl and Roxanne 2022). But such a proposal should dialogue with crucial aspects for bioethics, like privacy, voluntariness, and informed consent (Ausín and Andreu Martínez 2020).

As SDOH are highly contextual and based on cultural, social, and economic aspects that differ between places, it might not be enough to maximize representativity in global databases. The homogenization of community characteristics will always lead to possible biases if we use SDOH as a pattern of analysis. From this perspective, and to be fair with different realities all over the world, scalability is definitely in tension with disparities in local areas. If this be true, AI systems based on global datasets would need other procedures to adapt the results because they would fail to consider SDOH at a more than superficial level. There is a need to continue doing research to see if massive AI systems are the best option instead of local and smaller databases adapted to different cultural communities.

We have also found that ML solutions can be epidemiologically effective and, at the same time, ethically fraught because of design biases. The rapid development of DCT solutions has proven to accelerate the identification of infected people despite its ethical cost, raising worrying issues of lack of privacy, biased data, or socio-economic disparities. Our findings reiterate the lack of comparative studies and literature about the effectiveness and convenience of DCTApps during the pandemic outbreak, as previous research has concluded. This makes it more difficult to evaluate the success of the solutions and their cost in opportunity as well as to assess changes and improvements of this technology. Further research on this issue is required.

Finally, gender is never questioned as a possible bias on the results in published studies about biases in AI systems for COVID-19. Race, age, or socioeconomic status, on the contrary, appear more often as indicators in the data used in AI systems. Usually, biases are not due to a single attribute (e.g., gender) but to the combination of some of them (e.g., race, gender, poverty). That is why, instead of the classical single attribute fairness, there is a need for a multi-attribute solution, which follows the so-called intersectional approach. The intersectionality approach is useful to understand how gender intersects with other social identities (e.g., race, ethnicity, socioeconomic status, sexuality, or disability), increasing marginalization, social disadvantage, and bad healthcare outcomes (Kapilashrami and Hankivsky 2018; Figueroa et al. 2021). Biases in AI should be viewed through an intersectional lens because several of them occur together and in an overlapping way. This approach, vital to understanding SDOH and their relation to AI outcomes, has some difficulties. As Foulds et al. (2020) explain, data sparsity becomes a bigger problem when the number of dimensions or distinct values increase, leading to uncertainty in fairness measurements.

As datasets are imbalanced and can create unfair decisions to minority groups, there must be a counterbalance in which different inputs can be given different weights in the final prediction. For instance, Figueroa et al. (2021) explore how non-binary persons are under-represented in medical research, concluding that very few digital health interventions for non-binary individuals have been developed. As the authors states, “the absence of research on electronic health and mobile health in sexual and gender minority populations can lead to digital interventions being ineffective or even harmful for these groups, through biases in the data.” Although there are some papers and proposals on the theme in a general level (Foulds et al. 2020; Roy, Iosifidis, and Ntoutsi 2021), further research is required to develop an intersectional perspective in AI systems —regarding the conceptualization and the computational design—both in a general level and also specifically as developed for DCT and for PRP or medical triage to deal with any future COVID-19 pandemics that may come.

Our findings are in agreement with Ausín and Andreu Martínez (2020), who have found some ethical elements to take into consideration for DCT:

The security and safety of technological systems responds to the duty not to cause harm and to minimize harm, protecting individuals and groups.
The intervention must be proportional and beneficial given the severity of the situation.
The installation of the app must be voluntary and require people’s consent and not carry a penalty for non-acceptance.
4.The data must be pseudonymized to protect privacy.
Applications and other technologies must be available and accessible to everyone, regardless of economic or technological level.

There are some limitations to our study. First, we had some problems identifying AI from other algorithms or statistical and mathematical methods without AI because of a lack of clarity in the literature. Second, our search strategy may have overlooked some concepts related to SDOH or included them in the category. Third, the majority (thirteen out of twenty) of the included articles are narrative reviews (n = 10), rapid review (n = 1), or case studies (n = 2). Narrative reviews can be associated with selection bias (Pae 2015). However, these articles were selected because they addressed bias and other ethical issues more explicitly than other types of articles or empirical studies and because our aim was to identify current known biases in literature so future research can look for other kind of possible biases during the development and application of the AI support systems. Finally, since we restricted our review to studies published in English and Spanish, we might have missed relevant work published in other languages.

Recognizing these limitations, we hope that our scoping review can help to document the types and extent of biases actually present in specific AI algorithms for triage or PRP and DCT in the context of the COVID-19 pandemic.

## Conclusions

The analysis of previous literature shows that the main sources of biases identified in both triage or PRP and DCT AI systems for COVID-19 are mainly related to data source variability and inadequate data collection. In addition, ethical problems related to privacy, consent, and the lack of regulation have been identified in DCT Apps. Biases related to health disparities and SDOH are not the main topics of the studies but are in some way included, especially in narrative reviews of DCT Apps. Although there is some concern on the topic, a theoretical framework addressed to researchers and engineers would facilitate the comprehension and identification of potential biases in future technologies and their uses.
